# Ultrarare Coding Variants and Cognitive Function in Schizophrenia

**DOI:** 10.1001/jamapsychiatry.2022.2289

**Published:** 2022-08-17

**Authors:** Hugo D. J. Creeth, Elliott Rees, Sophie E. Legge, Charlotte A. Dennison, Peter Holmans, James T. R. Walters, Michael C. O’Donovan, Michael J. Owen

**Affiliations:** 1 MRC Centre for Neuropsychiatric Genetics and Genomics, Division of Psychological Medicine and Clinical Neurosciences, Cardiff University School of Medicine, Cardiff, United Kingdom

## Abstract

**Importance:**

Impaired cognitive function in schizophrenia is associated with poor functional outcomes, but the role of rare coding variants is unclear.

**Objective:**

To determine whether ultrarare constrained variants (URCVs) are associated with cognition in patients with schizophrenia.

**Design, Setting, and Participants:**

Linear regression was used to perform a within-case genetic association study of URCVs and current cognition and premorbid cognitive ability. A multivariable linear regression analysis of the outcomes associated with URCVs, schizophrenia polygenic risk score, polygenic risk score for intelligence and schizophrenia associated copy number variants on cognitive ability was performed. Exome sequencing data from 802 participants with schizophrenia were assessed for current cognition using the Measurement and Treatment Research to Improve Cognition in Schizophrenia Consensus Cognitive Battery and for estimated premorbid IQ using the National Adult Reading Test. Individuals were recruited from clinical and voluntary mental health services in the UK. Those with a diagnosis of intellectual disability or a neurological disorder known to affect cognition were excluded. Data collection occurred between 2007 and 2015. Data were analyzed between April 2020 and March 2022.

**Main Outcomes and Measures:**

Association between URCVs, current cognition, and current cognition adjusted for premorbid IQ.

**Results:**

Of the 802 participants, 499 (62%) were men and 303 (38%) were women; mean (SD) age at interview was 43.36 (11.87) years. Ultrarare constrained variants (n = 400) were associated with lower current cognition scores (β = −0.18; SE = 0.07; *P* = .005). In the univariable analysis, premorbid IQ was associated with URCVs (β = −0.12; SE = 0.05; *P* = .02) and partly attenuated the association with current cognition (β = −0.09; SE = 0.05; *P* = .08). Multivariable analysis showed that measured genetic factors combined accounted for 6.2% of variance in current cognition, 10.3% of variance in premorbid IQ, and supported outcomes of URCVs associated with current cognition independent of premorbid IQ (β = −0.10; SE = 0.05; *P* = .03).

**Conclusions and Relevance:**

The findings of this study suggest that URCVs contribute to variance in cognitive function in schizophrenia, with partly independent associations before and after onset of the disorder. Although the estimated effect sizes were small, future studies may show that the effect sizes will be greater with better annotation of pathogenic variants. Genomic data may contribute to identifying those at particularly high risk of cognitive impairment in whom early remedial or preventive measures can be implemented.

## Key Points

**Question:**

Are ultrarare constrained variants (URCVs) associated with reduced cognitive function in individuals diagnosed with schizophrenia?

**Findings:**

In this within-case genetic association study of 802 individuals with schizophrenia who had undergone exome sequencing and cognitive testing, significantly reduced cognitive function was found in individuals carrying URCVs.

**Meaning:**

This study found that URCVs were associated with reduced general cognitive function in schizophrenia; with better annotation of pathogenic variants, genomic data may contribute to identifying those with schizophrenia at particularly high risk of cognitive impairment in whom early remedial or preventative measures can be implemented.

## Teaser

This genetic association study assesses whether rare coding variants are associated with current cognitive function in patients with schizophrenia.

## Introduction

Schizophrenia displays considerable variation in clinical features, course, and outcome.^1^ It is also associated with variable impairments in cognitive function,^2^ and IQ is lower by approximately 1 SD relative to the general population.^3^ The nature of the association between cognitive function and schizophrenia is poorly understood but is of importance because there is a large body of evidence that cognitive impairment is associated with poor functional outcomes in work, independent living, and social integration.^2^

Schizophrenia is typically first diagnosed when psychosis becomes manifest, usually in late adolescence or early adulthood, but premorbid impairments in cognition frequently occur.^4^ People with schizophrenia have an average premorbid IQ of 0.5 SD lower than controls.^3^ The association of schizophrenia with various premorbid developmental deficits, including cognitive impairment, together with evidence for association with environmental exposures in utero and in early childhood, and, more recently, genetic evidence, support the idea that schizophrenia is, at least in part, a neurodevelopmental disorder.^5,6,7^ There is also evidence^3,8,9^ for a further decline in cognitive function after diagnosis, but it is not clear whether this decline reflects ongoing processes intrinsic to the pathophysiology of schizophrenia or secondary factors such as medication effects, comorbid physical illness, substance misuse, or ascertainment bias.

Schizophrenia is highly heritable and polygenic, with risk conferred by alleles across the frequency spectrum including common risk alleles,^10^ rare copy number variants (CNVs),^11,12^ and rare damaging coding variants.^13,14,15,16,17,18,19^ There is also clear evidence that the CNVs^20^ and genes associated with schizophrenia through both common^21^ and rare coding variants^17,22^ overlap with CNVs and genes implicated in childhood-onset neurodevelopmental disorders (NDDs).^13,15,17,19,20,21,22^

Cognitive function is moderately heritable in the general population and, similar to schizophrenia, is highly polygenic and affected by alleles across the frequency spectrum including common variants,^23^ CNVs^24,25^ and rare coding variation.^26^ Many of the common alleles that influence liability to schizophrenia also influence IQ in the general population,^23^ although it is unclear whether they also influence cognitive function in people with schizophrenia^27,28,29,30,31,32^ perhaps reflecting the modest samples sizes and power of these studies, differences in duration of illness at the time of testing and the nature of the cognitive tests used. Nevertheless, common alleles that are associated with higher intelligence in the general population are associated with better cognitive ability in individuals with schizophrenia.^27^

At the rare variant level, cognitive function in people with schizophrenia who are carriers of CNVs is, on average, approximately 0.5 to 1.0 SDs below that of noncarriers.^33^ Within people with schizophrenia, de novo protein truncating variants (PTVs) are more common in those with relatively poor school performance^14^ while the incidence of rare PTVs is higher in those with comorbid intellectual disability.^15^ Together, these findings are consistent with the hypothesis that rare coding variants may be associated with a higher risk of cognitive impairment in schizophrenia, although no study to date has investigated this in individuals with schizophrenia who have undergone quantitative assessment of cognitive function.

Given the association between cognitive function and functional outcome, it is important to understand the timing of, and mechanisms behind, cognitive impairment in schizophrenia to inform the design and implementation of interventions. Two recent studies^27,33^ of schizophrenia have examined genetic risk factors and timing, specifically premorbid and postonset cognitive function (referred to as current cognition hereafter). The first^33^ showed CNV carrier status was associated with substantially impaired current and premorbid cognitive function. The second^27^ found that common variant liability for IQ was associated with both current cognition and premorbid IQ, but the association with current cognition was largely explained by premorbid effects. In contrast, schizophrenia liability was associated only with current cognitive ability, and this association was independent of premorbid IQ. These findings suggest that common genetic variation that influences IQ in the general population and rare pathogenic CNVs contribute to premorbid cognitive impairment in schizophrenia, and that common schizophrenia risk alleles may be associated with further impairment after onset.

In the present study, we sought to assess whether rare coding variants are associated with current cognitive function in patients with schizophrenia, and to investigate the timing of any observed outcomes with respect to onset of the disorder. Unlike previous studies,^14,15^ we were able to examine cognition quantitatively across the range of cognitive abilities. Moreover, we also had estimates of premorbid IQ, allowing us to study for the first time to date the timing relative to disease onset at which genetic factors are apparent. In addition to exome sequencing data, we had CNV and single-nucleotide variations array data, which allowed us to investigate the combined and independent cognitive outcomes in schizophrenia associated with rare coding variants, CNVs, and common variants.

## Methods

### Sample and Phenotype Description

We included 873 participants prior to quality control (QC) from the Cardiff Cognition in Schizophrenia cohort,^12,34^ which consists of patients with a clinical diagnosis of schizophrenia. eMethods 1 in the Supplement includes a full sample description. Data collection occurred between 2007 and 2015. Data were analyzed between April 2020 and March 2022. This study was approved by the UK National Health Service, and written informed consent was obtained for all study participants. This study followed the Strengthening the Reporting of Genetic Association Studies (STREGA) reporting guideline for genetic association studies.

Current cognition was assessed using the Measurement and Treatment Research to Improve Cognition in Schizophrenia Consensus Cognitive Battery (MCCB) across 7 domains (eMethods 2 in the Supplement).^35^ Premorbid IQ was estimated using the National Adult Reading Test (NART).^36^ A full description of the cognitive assessments is available in eMethods 2 in the Supplement.

### Sequencing

A total of 472 samples were sequenced at the Broad Institute of MIT and Harvard University using the HiSeq X Platform (Illumina), and 401 samples were sequenced at Cardiff University using the HiSeq 3000/4000 Platform (Illumina). All sequencing data were processed using the same Genome Analysis Toolkit pipeline.^37^ Further sequencing details are presented in eMethods 3, eFigure 1, eFigure 2, and eTable 1 in the Supplement.

### Sequencing Quality Control

Samples were excluded if less than 70% of the exome target achieved 10X coverage (eMethods 3 in the Supplement) or if their sex inferred from the sequencing data did not match their recorded sex (eMethods 4 and eFigure 3 in the Supplement). We focused on individuals of European ancestry because there were insufficient numbers of participants of other ancestries to form informative substrata (eFigure 4 in the Supplement). Individuals were excluded to ensure that no samples were second degree or closer in relationship (eFigure 5 in the Supplement). In addition, samples were excluded if 1 or more of the hard filters described in eMethods 4 in the Supplement was applicable (eFigure 6 in the Supplement). In total, 71 cases were excluded (eTable 2 in the Supplement). Of the 802 individuals who remained after QC, 754 had measures of premorbid IQ, 762 of current cognition, and 721 for both. An overview of our genotype QC, variant QC, and variant annotation is available in eMethods 5 in the Supplement.

### Polygenic Risk Scores and Copy Number Variants

Standardized polygenic risk scores (PRS) for schizophrenia (SZ PRS) and intelligence (IQ PRS) were calculated using PRSice^38^ following a widely applied method^39^ using default parameters unless otherwise stated.^27^ Polygenic risk scores were adjusted for 5 principal components and based on single-nucleotide variants associated with a threshold of *P* ≤ .05 in the source genome-wide association study (eMethods 6 in the Supplement). Copy number variant calls were generated as detailed in a previous publication^33^ and in eMethods 6 in the Supplement.

### Study Design

We performed a within-case genetic association study of the association between the incidence of rare coding variants and cognitive ability. Recent studies have reported that evidence of selective constraint, at either the variant or gene level, is a feature associated with rare coding variants that contribute to impaired cognition in people with schizophrenia^15^ or autism spectrum disorder,^40^ as well as in the general population.^26^ Thus, in the current study, we postulated that ultrarare constrained variants (URCVs), defined as either PTVs in LoFI genes (genes with gnomAD probability of loss-of-function intolerance scores ≥0.9^41^) or damaging-missense variants (MPC ≥2)^42^ that are observed once in our sample and are not present in the gnomAD nonneuro data set, are associated with lower measures of current cognition in people with schizophrenia (eMethods 5 in the Supplement). We next investigated URCVs in terms of estimated premorbid IQ to assess whether these outcomes explained the associations between URCVs and current cognition.

### Statistical Analysis

Linear regression was used to test for association between cognition and the number of URCVs carried by each individual. We covaried for sex, age at interview, sequencing site, principal components 1 through 10, and the exomewide incidence of ultrarare synonymous variants. *R^2^*, which represents the proportion of phenotypic variance explained by the relevant models, was estimated from the multivariable linear models and univariable linear models. Statistical significance was determined as 2-sided *P* < .05. Further details of statistical analysis and study design are available in eMethods 7 in the Supplement.

## Results

### Ultrarare Constrained Variants

Of the 802 participants, 499 (62%) were men and 303 (38%) were women; mean (SD) age at interview was 43.36 (11.87) years. Consistent with our primary hypothesis, the incidence of URCVs was associated with lower current cognitive ability (Table 1), each variant associated with a reduction in performance of 0.18 SDs (current cognition: β = −0.18; SE = 0.07; *P* = .005; current cognition conditioned on premorbid IQ: β = −0.09; SE = 0.05; *P* = .08; and premorbid IQ: β = −0.12; SE = 0.05; *P* = .02). Effect sizes were robust to controlling for primary *DSM-IV* diagnosis (eResults and eTable 3 in the Supplement), or excluding PTVs considered low confidence by LOFTEE^41^ (eTable 4 in the Supplement). To explain this primary association signal, we tested the effect sizes on cognition conferred separately by ultrarare damaging missense variants (MPC ≥2) and ultrarare PTVs in LoFI genes, but found no differences between the 2 classes of mutation (eTable 5 in the Supplement).

**Table 1.** Ultrarare Constrained Variants and Cognitive Ability in Schizophrenia and Related Psychotic Disorders^a^

No. of Variants^b^	Cognitive measure^c^	Effect size (SE)^d^	*P* value
400	Current cognition	−0.18 (0.07)	.005
375	Current cognition (conditioned on premorbid IQ)^e^	−0.09 (0.05)	.08
392	Premorbid IQ	−0.12 (0.05)	.02

^a^ Ultrarare constrained variants (PTVs in loss-of-function intolerant genes and damaging missense variants [MPC ≥2]) were tested for association with premorbid IQ in 754 participants, 762 participants with current cognition and 722 participants with scores for both premorbid IQ and current cognition with schizophrenia and related psychotic disorders.

^b^ The differences in the number of variants are associated with the differences in sample size between participants with either premorbid IQ or current cognition scores.

^c^ The test refers to the dependent variable being tested using our linear regression model.

^d^ The effect size is the β estimate from the linear regression and corresponds to the difference in standardized cognition measure associated with each ultrarare constrained variant carried.

^e^ The test investigating current cognition when conditioned on premorbid IQ using our linear regression model.

### Timing and Cognition

After covarying for premorbid IQ, the effect size for URCVs and current cognition was substantially attenuated (β = −0.09; SE 0.05; *P* = .08) (Table 1). Similar results were obtained in a multivariable analysis that considered other classes of genetic variation (β = −0.10; SE 0.05; *P* = .03). The outcome of URCVs associated with current cognition covarying for premorbid IQ was not attributable to duration of illness or age at onset of schizophrenia (eTable 6 in the Supplement).

The results suggest that URCVs are factors in cognitive function after the onset of illness or have only premorbid outcomes, but these may affect cognitive domains indexed by MCCB but not NART. To examine the latter hypothesis, we investigated URCVs and MCCB domains but found similar effect sizes across all domains, the exception being social cognition in which no effect size was apparent (eTable 7 in the Supplement). Moreover, the domain that had the highest Pearson correlation coefficient with NART (ie, working memory) was also the current cognition domain that had the highest effect size with URCVs. Together these findings may counter the suggestion of URCVs having restricted premorbid effects on domains of cognition that are not indexed by NART (eTable 7 in the Supplement).

### URCVs in NDD Genes and Non-NDD Genes

As an exploratory analysis, we compared cognitive ability in schizophrenia with URCVs in NDD genes with those in genes not associated with NDD (non-NDD genes), recognizing that the latter group will contain genes that have yet to be implicated in NDDs. While the point estimates of the effect sizes were substantially greater for URCVs in NDD genes than in non-NDD genes, the differences were not statistically significant (Table 2).

**Table 2.** Association Between Cognitive Ability and URCVs in NDD Genes^a^

Cognitive measure	Constrained variant set^b^	No. of Variants^c^	Effect size (SE)^d^	*z* Test
Premorbid IQ	In NDD genes	51	−0.26 (0.14)	0.43
	Non-NDD genes	341	−0.1 (0.05)	
Current cognition	In NDD genes	52	−0.36 (0.18)	0.42
	Non-NDD genes	348	−0.16 (0.07)	

Abbreviations: NDD, neurodevelopmental disorder; URCVs, ultrarare constrained variants.

^a^ The outcomes on cognitive ability of URCVs in known NDD genes was compared with the outcomes of URCVs in non-NDD genes. These variants were evaluated for association with premorbid IQ in 754 participants and current cognition in 762 participants with schizophrenia and related psychotic disorders. A *z* test was used to compare the variance in the effect sizes between URCVs in NDD and non-NDD genes.

^b^ The independent variable being tested using our linear regression model.

^c^ The differences in the number of variants is associated with the differences in sample size between participants with either premorbid IQ or current cognition scores.

^d^ The effect size is the β estimate from the linear regression, and corresponds to the difference in standardized cognition measure associated with each URCV carried.

### URCVs, CNVs, Schizophrenia PRS, IQ PRS, and Cognitive Function

In addition, we performed multivariable analyses on a subset of 648 individuals who had data on URCVs, CNVs (eTable 8 in the Supplement), SZ PRS, and IQ PRS. The estimated effect sizes (Table 3) were similar to those obtained from the univariable models (eTable 9 in the Supplement) (for example, in the multivariable analysis, the URCV effect size on premorbid IQ was β = −0.14; SE, 0.05; *P* = .01; in the univariable analysis, the URCV effect size on premorbid IQ was β = −0.12; SE, 0.05; *P* = .02), which is broadly consistent with the different classes of variant acting independently on cognition. After conditioning on premorbid IQ, all effect sizes on current cognition were attenuated except for the SZ PRS (for example, in the multivariable analysis the URCV, effect size on current cognition without covarying for premorbid IQ was β = −0.19; SE = 0.07; *P* = .005; in the multivariable analysis the URCV, effect size on current cognition when covarying for premorbid IQ was β = −0.10; SE = 0.05; *P* = .03) (Table 3 and Table 4).

**Table 3.** Multivariable Analyses of Cognition of URCVs, SZ PRS, IQ PRS, and CNV Carrier Status^a^

Cognitive measure and genetic component	Multivariable analysis^b^		Multivariable analysis covarying for premorbid IQ^c^	
	Effect size (SE)^d^	*P* value	Effect size (SE)	*P* value
**Premorbid IQ**				
URCVs	−0.14 (0.05)	.01	NA	NA
SZ PRS	0.03 (0.04)	.41	NA	NA
IQ PRS	0.31 (0.04)	<.001	NA	NA
CNV	−0.73 (0.27)	.01	NA	NA
**Current cognition**				
URCVs	−0.19 (0.07)	.005	−0.10 (0.05)	.03
SZ PRS	−0.07 (0.05)	.12	−0.07 (0.04)	.06
IQ PRS	0.29 (0.05)	<.001	0.09 (0.04)	.03
CNV	−0.68 (0.36)	.06	−0.27 (0.30)	.36

Abbreviations: CNV, copy number variant; IQ PRS, IQ polygenic risk score; NA, not applicable; SZ PRS, schizophrenia polygenic risk score; URCVs, ultrarare constrained variants.

^a^ URCVs, SZ PRS, IQ PRS, and CNV carrier status were tested within a multivariable linear regression model to evaluate outcomes associated with premorbid IQ in 679 participants and current cognition in 648 participants with schizophrenia or related psychotic disorders.

^b^ All the genetic components being included within our linear regression model as independent variables.

^c^ The multivariable analysis including premorbid IQ as a covariable within our linear regression model.

^d^ The effect size is the β estimate from the linear regression and corresponds to the difference in standardized cognition measure associated with each URCV carried or an increase of 1 SD of the PRS.

**Table 4.** Variance Explained by URCVs, SZ PRS, IQ PRS, and CNVs^a^

Cognitive measure and model^b^	*R^2^* of model	Variance explained by genetic component, %^c^
**Premorbid IQ**		
Baseline^d^	0.033	NA
SZ PRS + baseline	0.035	0.2
IQ PRS + baseline	0.124	9.1
CNV + baseline	0.041	0.8
URCVs + baseline	0.034	0.1
All genetic + baseline	0.136	10.3
**Current cognition**		
Baseline	0.151	NA
SZ PRS + baseline	0.157	0.6
IQ PRS + baseline	0.197	4.6
CNV + baseline	0.155	0.4
URCVs + baseline	0.153	0.2
All genetic + baseline	0.213	6.2
Baseline (including premorbid IQ)	0.432	NA
SZ PRS + baseline (including premorbid IQ)	0.441	0.9
IQ PRS + baseline (including premorbid IQ)	0.442	1.0
CNV + baseline (including premorbid IQ)	0.433	0.1
URCVs + baseline (including premorbid IQ)	0.433	0.1
All genetic + baseline (including premorbid IQ)	0.448	1.6

Abbreviations: CNV, copy number variant; IQ PRS, IQ polygenic risk score; NA, not applicable; SZ PRS, schizophrenia polygenic risk score; URCVs, ultrarare constrained variants.

^a^ *R^2^* from both the multivariable linear models and univariable linear models represents the proportion of phenotypic variance explained by the relevant models including baseline in 648 samples. The proportions of variance of the cognitive measure after correction for the baseline variables explained by each genetic component are their values in the Table divided by (1−*R^2^* [baseline]).

^b^ Model refers to the linear regression model.

^c^ Variance explained of the genetic component is the *R^2^* of the relevant model minus the *R^2^* of the model containing the baseline covariates alone.

^d^ The baseline covariates that are included in each model were age at interview, sex, sequencing site, synonymous variants, and principal components 1 through 10. Premorbid IQ is included in the baseline for all analyses as indicated.

All measured genetic factors accounted for 6.2% of the variance in current cognition and 10.3% of the variance in premorbid IQ (Table 4). After controlling for premorbid IQ, all measured genetic factors accounted for 1.6% of the variance in current cognition.

## Discussion

We have investigated the contribution of genetics to variation in cognitive ability in individuals with schizophrenia. We focused primarily on URCVs, a class of mutation that contributes to risk of schizophrenia,^13,14,15,16,17,18,19^ and which is particularly enriched in people with schizophrenia who have poor school performance^14^ and comorbid intellectual disability.^15^ We found that URCVs contribute more generally to variance in current cognitive function in schizophrenia rather than simply being enriched in patients with intellectual disability. Our finding that the distribution of premorbid IQ and current cognition scores in URCV carriers largely overlaps that of noncarriers (eFigure 7 in the Supplement) supports this conclusion. Each URCV was associated on average with 0.18 SD lower current cognitive performance as indexed by the Measurement and Treatment Research to Improve Cognition in Schizophrenia composite score.

Those who develop schizophrenia frequently exhibit impairments in cognition before diagnosis.^3,4^ These deficits appear to have their origins in childhood,^3,43,44,45^ clearly cannot be a consequence of manifest disorder and, together with associations with other premorbid developmental deficits and environmental exposures, support the idea that schizophrenia is, at least in part, a neurodevelopmental disorder.^5,6,7^ Given that URCVs in schizophrenia are enriched in genes implicated in childhood neurodevelopmental disorders,^13,14,15,17,19^ and some of the same mutations that occur in schizophrenia also occur in NDDs,^22^ we hypothesized that the outcomes of URCVs associated with current cognition would be the result of premorbid effects. This hypothesis was partly confirmed, URCVs being associated with a 0.12 SD reduction in the premorbid IQ (Table 1), but there was evidence for additional effects on current cognition after conditioning on NART. We acknowledge that this issue, which has potentially important implications for the timing of remedial interventions, requires further investigation in larger samples.

The residual outcomes might reflect direct intrinsic effects of schizophrenia pathophysiology, with neurodevelopmental processes playing out over time into early adulthood.^45^ Alternatively, or in addition, they may be outcomes of active symptomatology or indirect consequences of illness, such as medication effects, lifestyle factors, poverty, and other aspects of social disadvantage. To the extent that carriers and noncarriers of URCVs have forms of schizophrenia that are, at least for now, essentially indistinguishable, it might be supposed that indirect effects of the illness are likely to be equally shared between the 2 groups. It would then follow that their effects should not differ between carriers and noncarriers of URCVs. However, given that poor cognition is associated with worse outcomes, it seems reasonable to suggest that those with poorer cognitive ability at the outset, including URCV carriers, may be further disadvantaged by greater exposure to potentially toxic secondary factors and consequently more likely to show current cognition impairments. Similar arguments can be invoked to account for our observation that there was a residual effect of IQ PRS on current cognition when conditioning on NART.

A multivariable analysis of URCV along with other types of genomic variation previously associated with schizophrenia and/or cognition were consistent with the different classes of variant acting independently on cognition. The largest effect sizes on both current and premorbid IQ came from CNVs followed by IQ PRS and then URCVs. The SZ PRS showed weak evidence for association to current cognition in the univariable analysis (eTable 9 in the Supplement) but not in the multivariable analysis (Table 3). As for URCVs, CNVs and IQ PRS associations with current cognition were markedly attenuated after controlling for premorbid IQ, a finding previously noted for IQ PRS in the present sample in a univariable analysis.^27^

At a population level, variance explained allows for an assessment of the contribution of different classes of variant that considers differences in allele frequencies and effect sizes. The multivariable model indicated that the genetic factors measured in this study account for a total of 10.3% of the variance in premorbid IQ and 6.2% of the variance in current cognition, the latter decreasing to 1.6% of the variance after conditioning on premorbid IQ (Table 4).

Each URCV was associated with a 0.18 SD lower MCCB composite score, equivalent to a reduction of only 2.7 in IQ points. When considering this in relation to the effect sizes found with the other classes of mutation on cognition, it is important to note that the discovery of potentially relevant rare variation is at an early stage, and studies of much larger samples are warranted. Moreover, in the case of URCVs, the estimated effect sizes of alleles of true effect will have been reduced by the inclusion of many alleles that have no effect on either schizophrenia liability or cognition. Consistent with this idea, URCVs that are likely to be enriched for those with true effects by virtue of being located in genes associated with neurodevelopmental disorders yielded a larger point estimate for their effect size than the same classes of mutations in non-NDD genes (Table 2) and were similar in effect size to those for IQ PRS. We acknowledge the estimated effect size of URCVs in NDD genes was not significantly greater than variants in the non-NDD set, possibly reflecting the relatively small number of URCVs in NDD genes, but these findings support the idea that better annotation and classification of pathogenic URCVs will increase the estimated effect size of this class of alleles, while more complete discovery will increase its contribution to the variance explained. This situation may suggest that, although URCVs are by definition uncommon, it may be possible to use them to identify a small subgroup of individuals with early signs of schizophrenia or with increased risk of schizophrenia, who are at higher risk of subsequent cognitive decline and in whom early remedial or preventative measures can be implemented. The discovery of rare risk alleles associated with cognitive decline might also help to implicate areas of biology that are important in the impairments in cognitive function that are seen more generally in schizophrenia and which affect functional outcomes.

### Strengths and Limitations

To our knowledge, this study is first to evaluate the association between URCVs and quantitative measures of current cognition in people with schizophrenia and to examine timing of the outcomes in the context of all known relevant classes of genomic variation. Limitations are that NART is an indirect measure of premorbid IQ, although this limitation is mitigated somewhat by work showing it to be strongly correlated with direct measures of premorbid IQ.^46^ Our study was focused on individuals of European ancestry because sufficient samples from cases of individuals with non-European ancestry were not available, but we expect the findings from URCVs and CNVs in terms of cognition in schizophrenia are more likely to generalize to individuals with non-European ancestries than the outcomes associated with IQ PRS, which was generated from European ancestry–based IQ genome-wide association study data.

## Conclusions

Results of this study suggest that URCVs were associated with cognitive impairment in schizophrenia, and we found evidence they may independently exert effects after onset of the disorder as well as premorbidly. In our study, the estimated effect sizes were small, but future studies may find that the effect sizes will be greater with better annotation of pathogenic variants. As findings from other studies accrue, we can expect them to inform the use of genomic data for identifying those individuals with, or at high risk of developing, schizophrenia who are particularly likely to develop subsequent cognitive impairment and in whom early remedial or preventative measures can be implemented.

## Notes

Supplement. **eMethods 1.** Sample Description and Psychiatric Assessment **eMethods 2.** Assessment of Cognition **eMethods 3.** Sequencing and Sequencing Coverage **eFigure 1.** Proportion of Bases Across the Full Exome Target That Were Sequenced at a Depth ≥10X **eFigure 2.** Sample Quality Metrics for Variants Within Consensus and Non-Consensus Target Regions **eTable 1.** Comparison of Ultra-Rare Coding Variants Between Sequencing Sites **eMethods 4.** Sample Quality Control (QC) **eFigure 3.** Somalier Inferred Sex Checks **eFigure 4.** Principal Components Analysis of CardiffCOGS Samples to Impute Ancestry **eFigure 5.** Sample Relatedness as Assessed Using the PC-Relate Method **eFigure 6.** Distributions of Variant Metrics for All CardiffCOGS Samples **eTable 2.** Summary of QC Sample Exclusions **eMethods 5.** Variant and Genotype QC and Variant Annotation **eMethods 6.** Polygenic Risk Scores and Copy Number Variants **eMethods 7.** Study Design and Statistics **eFigure 7.** Density Plots Showing the Distribution of Premorbid IQ and Current Cognition Scores for Carriers and Non-Carriers of Ultra-Rare, Constrained Variants
eResults
**eTable 3.** Association Between URCVS and Cognition When Covarying for Diagnosis **eTable 4.** Evaluating the Impact of Removing LOFTEE Low-Confidence Ptvs on the Association Between URCVS and Cognition in Schizophrenia **eTable 5.** Independent Effects of Damaging Missense Variants (MPC≥2) and Ptvs on Cognition **eTable 6.** Effects of Duration of Disorder and Age at Onset on Current Cognition Corrected for Age at Interview **eTable 7.** Specific Domains of Current Cognition and Their Association With URCVS **eTable 8.** Schizophrenia Associated Copy Number Variants Identified in the CardiffCOGS Cohort **eTable 9.** Univariable Analyses for the Independent Impact on Cognition of URCVS, Schizophrenia (SZ), IQ PRS and CNV Carrier Status
eReferences
