# Supplementary material for: Ultrarare Coding Variants and Cognitive Function in Schizophrenia
Source: JAMA Psychiatry. 2022 Aug 17;79(10):963–70. doi: 10.1001/jamapsychiatry.2022.2289 (PMC9386603; doi:10.1001/jamapsychiatry.2022.2289)
Supplement: Supplement. — eMethods 1. Sample Description and Psychiatric Assessment eMethods 2. Assessment of Cognition eMethods 3. Sequencing and Sequencing Coverage eFigure 1. Proportion of Bases Across the Full Exome Target That Were Sequenced at a Depth ≥10X eFigure 2. Sample Quality Metrics for Variants Within Consensus and Non-Consensus Target Regions eTable 1. Comparison of Ultra-Rare Coding Variants Between Sequencing Sites eMethods 4. Sample Quality Control (QC) eFigure 3. Somalier Inferred Sex Checks eFigure 4. Principal Components Analysis of CardiffCOGS Samples to Impute Ancestry eFigure 5. Sample Relatedness as Assessed Using the PC-Relate Method eFigure 6. Distributions of Variant Metrics for All CardiffCOGS Samples eTable 2. Summary of QC Sample Exclusions eMethods 5. Variant and Genotype QC and Variant Annotation eMethods 6. Polygenic Risk Scores and Copy Number Variants eMethods 7. Study Design and Statistics eFigure 7. Density Plots Showing the Distribution of Premorbid IQ and Current Cognition Scores for Carriers and Non-Carriers of Ultra-Rare, Constrained Variants eResults eTable 3. Association Between URCVS and Cognition When Covarying for Diagnosis eTable 4. Evaluating the Impact of Removing LOFTEE Low-Confidence Ptvs on the Association Between URCVS and Cognition in Schizophrenia eTable 5. Independent Effects of Damaging Missense Variants (MPC≥2) and Ptvs on Cognition eTable 6. Effects of Duration of Disorder and Age at Onset on Current Cognition Corrected for Age at Interview eTable 7. Specific Domains of Current Cognition and Their Association With URCVS eTable 8. Schizophrenia Associated Copy Number Variants Identified in the CardiffCOGS Cohort eTable 9. Univariable Analyses for the Independent Impact on Cognition of URCVS, Schizophrenia (SZ), IQ PRS and CNV Carrier Status eReferences [file jamapsychiatry-e222289-s001.docx]

Supplemental Online Content

Creeth HDJ, Rees E, Legge SE, et al. Ultrarare coding variants and cognitive function in schizophrenia. *JAMA Psychiatry.* Published online August 17, 2022. doi:10.1001/jamapsychiatry.2022.2289

**eMethods 1.** Sample Description and Psychiatric Assessment

**eMethods 2.** Assessment of Cognition

**eMethods 3.** Sequencing and Sequencing Coverage

**eFigure 1.** Proportion of Bases Across the Full Exome Target That Were Sequenced at a Depth ≥ 10X

**eFigure 2.** Sample Quality Metrics for Variants Within Consensus and Non-Consensus Target Regions

**eTable 1.** Comparison of Ultra-Rare Coding Variants Between Sequencing Sites

**eMethods 4.** Sample Quality Control (QC)

**eFigure 3.** Somalier Inferred Sex Checks

**eFigure 4.** Principal Components Analysis of Cardiff COGS Samples to Impute Ancestry

**eFigure 5.** Sample Relatedness as Assessed Using the PC-Relate Method

**eFigure 6.** Distributions of Variant Metrics for All Cardiff COGS Samples

**eTable 2.** Summary of QC Sample Exclusions

**eMethods 5.** Variant and Genotype QC and Variant Annotation

**eMethods 6.** Polygenic Risk Scores and Copy Number Variants

**eMethods 7.** Study Design and Statistics

**eFigure 7.** Density Plots Showing the Distribution of Premorbid IQ and Current Cognition Scores for Carriers and Non-Carriers of URCVs

**eResults**

**eTable 3.** Association Between URCVs and Cognition When Covarying for Diagnosis

**eTable 4.** Evaluating the Impact of Removing LOFTEE Low-Confidence PTVs on the Association Between URCVs and Cognition in Schizophrenia

**eTable 5.** Independent Effects of Damaging Missense Variants (MPC ≥ 2) and Ptvs on Cognition

**eTable 6.** Effects of Duration of Disorder and Age at Onset on Current Cognition Corrected for Age at Interview

**eTable 7.** Specific Domains of Current Cognition and Their Association With URCVs

**eTable 8.** Schizophrenia Associated Copy Number Variants Identified in the Cardiff COGS Cohort

**eTable 9.** Univariable Analyses for the Independent Impact on Cognition of URCVs, Schizophrenia (SZ), IQ PRS and CNV Carrier Status

**eReferences**

This supplemental material has been provided by the authors to give readers additional information about their work.

**eMethods 1**

***Sample description***

The Cardiff COGS cohort consists of patients with a clinical diagnosis of schizophrenia recruited from out-patient, in-patient and voluntary sector mental health services in the UK. Participants were excluded if they had a diagnosis of intellectual disability, or a known diagnosis of a neurological disorder known to affect cognitive functioning.

***Psychiatric assessment***

Participants were interviewed using the Schedules for Clinical Assessment in Neuropsychiatry (SCAN) ^1,2^. Trained psychiatrists or psychology graduates completed the clinical ratings and cognitive assessments under the supervision of the study principal investigator (J.T.R.W.). Best-estimate lifetime diagnosis was assigned using DSM-IV criteria ^3^ based on the SCAN interview and a vignette summary of the medical records. Participants had the following DSM IV diagnoses: schizophrenia (597), schizoaffective disorder depressive type (135), schizoaffective disorder bipolar type (72) and other non-affective psychotic disorders (69); there was good interrater reliability for diagnosis ^4^. Participants were aged 17 to 82 years.

**eMethods 2**

***Assessment of cognition***

Participants were assessed for current and estimated premorbid cognition. Current cognition was assessed using the Measurement and Treatment Research to Improve Cognition in Schizophrenia (MATRICS) Consensus Cognitive Battery (MCCB) across 7 domains (Processing Speed, Attention Vigilance, Working Memory, Verbal Learning, Visual Learning, Reasoning and Problem Solving and Social Cognition) ^5^. Following procedures in the MCCB manual ^6^, domain *z scores* were derived based on the mean and standard deviations of 103 healthy controls who were recruited as part of the study. A MATRICS cognitive composite z-score was calculated as described previously ^7^. As recommended in the MCCB Manual we used Sequential Regression Multiple Imputation to impute missing raw scores prior to calculating a composite z score for all participants who had data available for at least 5 of the 7 cognitive domains ^4^. The MATRICS composite z-score was used in this study as a measure of general cognition at the time of assessment (which we term *current cognition*). *Premorbid IQ* was estimated using the National Adult Reading Test (NART) ^8^.

**eMethods 3**

***Sequencing***

Exome sequencing data were generated across two sites: 472 samples were sequenced at the Broad Institute of MIT and Harvard University using the Illumina HiSeq X platform and Nextera DNA Exome capture kit, and 401 samples were sequenced at Cardiff University using the Illumina HiSeq 3000/4000 SBS Kit and Nextera DNA Exome capture kit. Raw sequencing reads from all samples were processed using the same GATK best practice pipeline ^9^. Sequence reads were aligned to the human genome reference build 37 (GRCh37) using the Burrow–Wheeler Aligner (bwa) v0.7.15 ^10^. Variants were called using GATK haplotype caller (version 3.4).

***Sequencing coverage***

Mosdepth ^11^ was used to calculate sequencing coverage across all coding regions and for each individual exon targeted in the Nextera capture kit. Samples were excluded if less than 70% of the exome target achieved 10X coverage. To control for differences in sequencing coverage across the sequencing sites (**eFigure 1**), analyses were restricted to consensus target regions (**eFigure 2**), defined as exons with a mean coverage 10X in samples sequenced at each site; of 214,024 target exons (45.3 Mb), 191,805 (40.9 Mb) met this criterion, retaining 90% of exons.

**eFigure 1:** **Proportion of bases across the full exome target that were sequenced at a depth ≥ 10X**.

Each dot shows the coverage for a sample sequenced either at the Broad Institute of MIT and Harvard Universities (red) or at Cardiff University (blue).

**eFigure 2:** **Sample quality metrics for variants within consensus and non-consensus target regions.**

To control for differences in sequencing coverage across the two sequencing sites (eFigure 1), analyses were restricted to consensus target regions (defined as exons with a mean coverage ≥10X in samples sequenced at each site). **A, B, C**: Show metrics for TiTv Ratio, Het/Hom Ratio and the number of novel SNPs carried by each sample in the non-consensus target regions (i.e. exon targets that did not achieve a mean coverage ≥10X in either the Broad or Cardiff sequenced samples). **D, E, F**: Show metrics for TiTv Ratio, Het/Hom Ratio and the number of novel SNPs carried by each sample when restricting to the consensus target regions. By restricting to consensus targets, sample metrics across the two sequencing sites followed more comparable distributions.

***
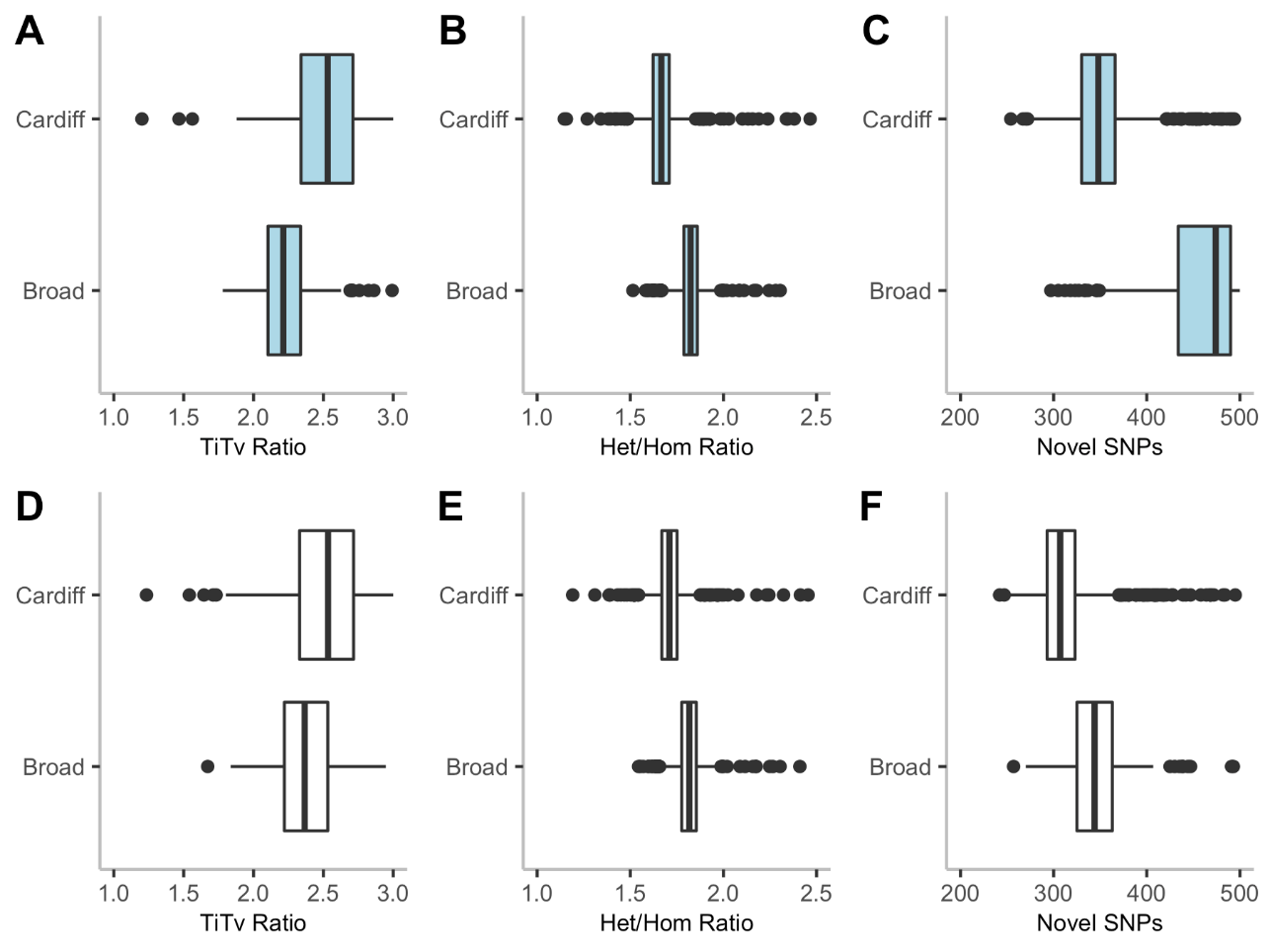
***

To control for differences in sequencing quality and coverage between samples sequenced at the Broad Institute and Cardiff University, our analyses included covariates for the exome-wide rate of ultra-rare synonymous variants and a binary variable for sequencing site (Broad or Cardiff, see methods for further details). As an additional analysis to confirm that our QC procedure minimised the effect of these sequencing differences on the number of rare mutations carried by each individual, we compared the rate of ultra-rare synonymous variants, missense variants (MPC > 2) and PTVs between samples sequenced at the Broad Institute and Cardiff University, and found no significant difference between these groups (**eTable 1**).

| **eTable 1: Comparison of ultra-rare coding variants between sequencing sites*.*** We performed a logistic regression to compare the rate of ultra-rare synonymous variants, missense variants (MPC > 2) and PTVs between samples sequenced at the Broad Institute and Cardiff University. | | | | |
| --- | --- | --- | --- | --- |
| **Test** | **Variant Type** | **Effect Size** | **S**E | ***P-Value*** |
| Sequencing Site  (Broad vs Cardiff) | MPC ≥ 2 | -0.03 | 0.13 | 0.85 |
|  | PTVs | -0.24 | 0.16 | 0.15 |
|  | Synonymous Variants | -0.05 | 0.03 | 0.09 |

**eMethods 4**

***Sample Quality Control (QC)***

Ancestry and sex imputation

﻿*Somalier* ^12^ was used to predict the ancestry and impute the sex of each sample. Sex was inferred from informative variants on the X and Y chromosomes, and from the rates of heterozygous and homozygous calls on the X chromosome (**eFigure 3**). Individuals were excluded if their inferred sex did not match their recorded sex.

**eFigure 3:** **Somalier inferred sex checks.**

*
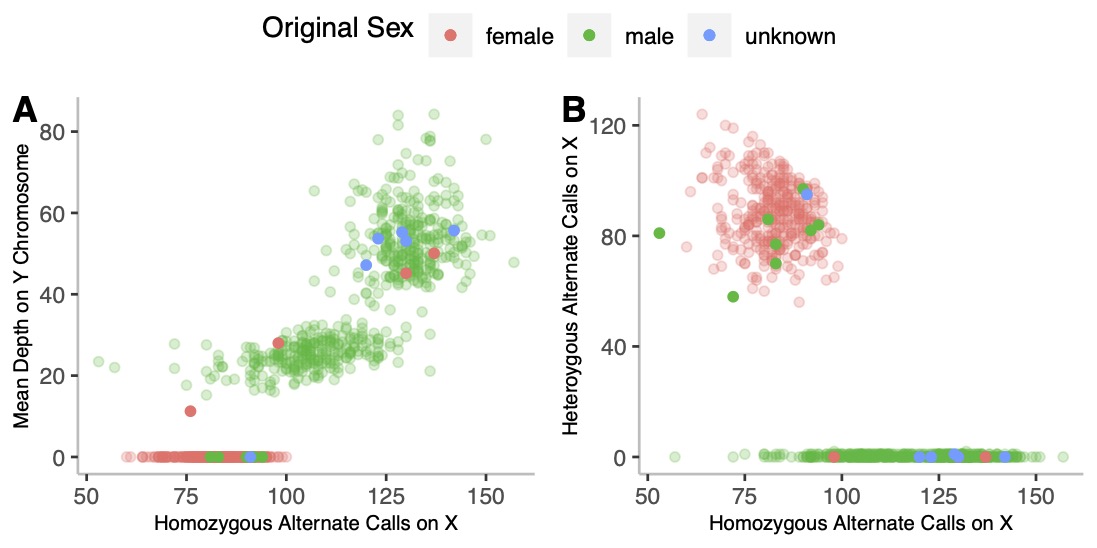
*The somalier sex check is based upon informative variants on the X and Y chromosome. Each point is a sample coloured as red if the sample was originally recorded as being female and green if it is recorded as male. **A**. For each sample, the x-axis shows the number of homozygous alternate variant calls, and the y-axis shows the mean sequencing depth on the Y chromosome. **B**. For each sample, the number of homozygous alternate variant calls is shown on the x-axis, and the number of heterozygous variant calls is shown on the y-axis. Samples that were originally indicated as one sex but group with the opposite sex were excluded.

Ancestry was inferred using Principal Components Analysis (PCA) by comparing our samples with samples of known ancestry from the thousand genomes project (1KG). In the present study, we focused on individuals of European ancestry as there are insufficient participants of other ancestries to form informative substrata. We excluded samples that fell 3 standard deviations or more from the mean of PCs 1 and 2 from the 1KG European samples (**eFigure 4**).

**eFigure 4:** **Principal components analysis of Cardiff COGs samples to impute ancestry.**

Ancestry was inferred using Principal Components Analysis (PCA) by comparing our samples with samples of known ancestry from the thousand genomes project (1KG). We focused our analysis on individuals of European (EUR) ancestry, due to there being insufficient participants from other ancestries (African – AFR, Amerindian- AMR, East Asian – EAS, South Asian – SAS) to form informative substrata. Samples were excluded if they fell 3 standard deviations or more from the mean of PCs 1 and 2 from the 1KG European samples (included in plot as lighter transparent colours).


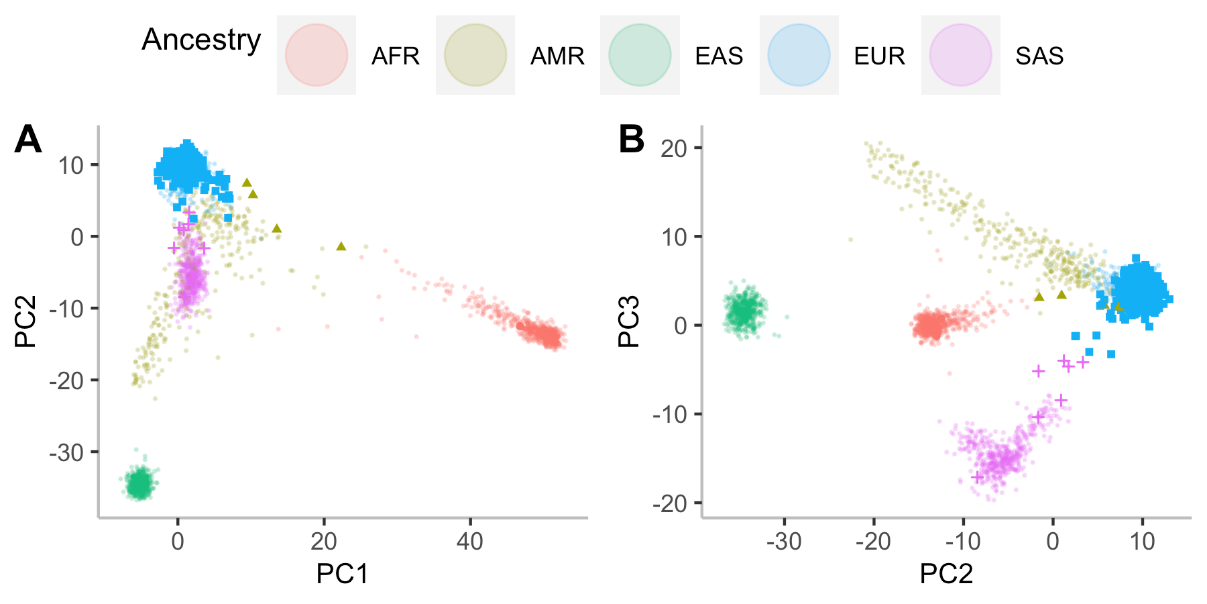


Relatedness

The PC-Relate method was applied in Hail (https://hail.is/docs/0.2/index.html) to estimate pairwise kinship coefficients (Φ_ij_) between all pairs of samples ^13^ using LD-pruned SNPs (max r^2^ < 0.1) with minor allele frequency (MAF) > 0.05 and call rate > 0.98. The first 5 PCs were included to correct for population structure in the kinship calculation. Pairs of individuals where Φ_ij_ **≥** 0.45 were considered duplicates or monozygotic twins, those with 0.2 < Φ_ij_ < 0.3 were considered first-degree relatives, and those with 0.1 < Φ_ij_ < 0.2 were considered second-degree relatives. We pruned clusters of related individuals to ensure that no two samples were second-degree, or closer in relationship (**eFigure 5)**.

**eFigure 5:** **Sample relatedness as assessed using the PC-relate method.**

Scatter plots show the estimated kinship coefficients against estimated proportion of alleles that have IBD0 for each pair of individuals.

**
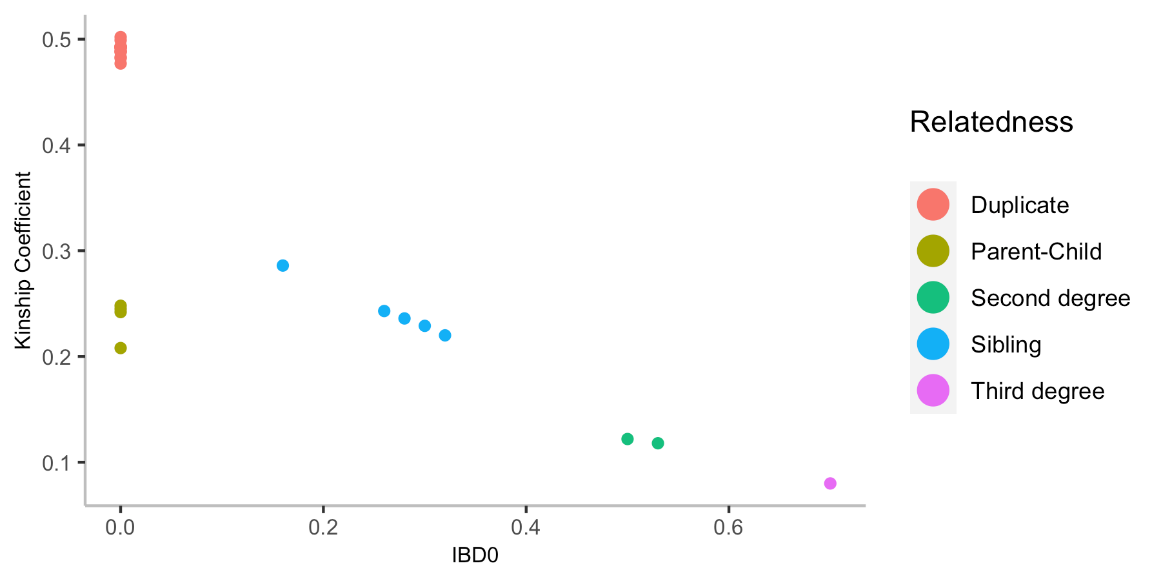
**

Hard Filters

We used Hail’s sample_qc function to generate the following sample metrics from the raw variant calls that were observed in the consensus target regions (described above in *Sequencing coverage,* ***eMethods 3****)*: call rate (callRate), number of heterozygous calls (nHet), number of homozygous calls (nHomVar), number of non-reference calls (nNonRef), number of deletions (nDeletion), number of insertions (nInsertion), number of singleton calls (nSingleton), number of SNPs (nSNPs), heterozygous-homozygous call ratio (rHetHomVar), transition-transversion ratio (rTiTv), and insertion-deletion (rInsertionDeletion). Sample metrics across the two sequencing sites followed comparable distributions.

Based on the observed distributions (**eFigure 6**), we applied the following filters to exclude low quality samples and individual outliers: callRate < 0.9, nDeletion < 500 | nDeletion > 700, nHet < 12500| nHet > 15000, nHomVar < 7500 | nHomVar > 9500, nInsertion < 550 | nInsertion > 750, nNonRef < 20000 | nNonRef > 25000, nSingleton < 10 | nSingleton > 400, nSNP < 27500 | nSNP > 32500, rHetHomVar < 1.3 | rHetHomVar > 1.9, rInsertionDeletion < 0.9 | rInsertionDeletion > 1.3, and rTiTv < 2.8 | rTiTv > 3.1. The hard filter QC removed a total of 18 individuals who were outliers for one or more of the above metrics.

**eFigure 6:** **Distributions of variant metrics for all Cardiff COGs samples.**

**
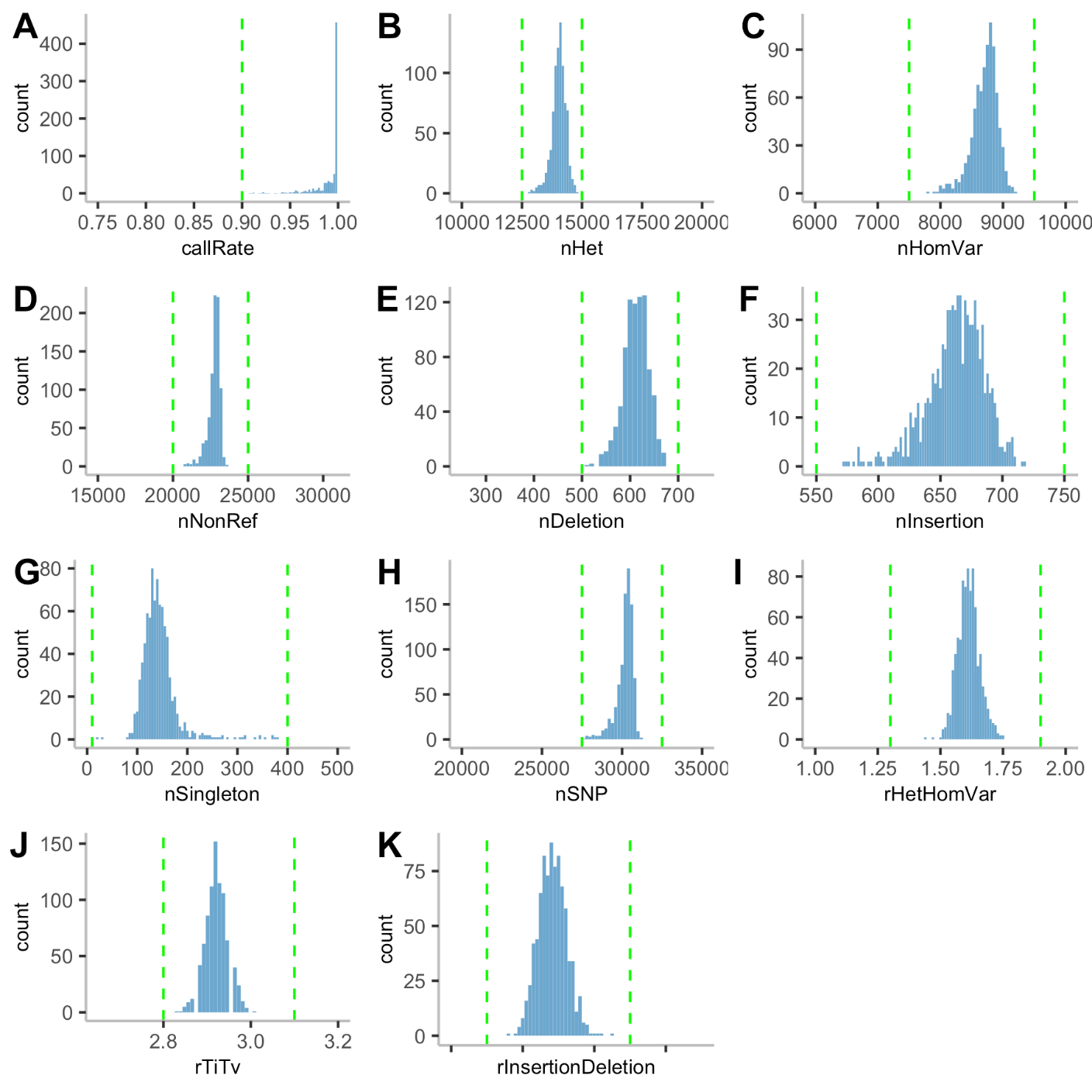
**Histograms showing the distributions of sample metrics used in the hard filter QC. The metrics are derived from variants observed in the consensus target regions. The green dotted lines indicate the thresholds used to filter samples from the analysis.

Summary of sample QC

Across all sample QC measures, we excluded 71 cases (**eTable 2**), and the final number of samples taken forward in our analysis was 802 individuals.

| **eTable 2:** **Summary of QC sample exclusions**  A total of 71 samples were excluded from our Cardiff COGs cohort after QC. These samples were excluded on the basis that they failed QC for sex, ancestry, relatedness, duplicates and hard filters. The final number taken forward for the analysis was 802 individuals with both sequencing and phenotype data. | | | | | | | | |
| --- | --- | --- | --- | --- | --- | --- | --- | --- |
| **Sequencing Site** | **N samples pre-QC** | **Sex Check** | **Ancestry** | **Relatedness** | **Duplicates** | **Hard filters** | **N samples post-QC** |  |
| **Broad** | 472 | 6 | 15 | 1 | 0 | 6 | 444 |  |
| **Cardiff** | 401 | 3 | 8 | 7 | 13 | 12 | 358 |  |
| **Totals** | **873** | **9** | **23** | **8** | **13** | **18** | **802** |  |

**eMethods 5**

***Variant and genotype QC***

Variants were filtered in Hail v0.1 (<https://github.com/hail-is/hail>) using the GATK Variant Quality Score Recalibration (VQSR) information available in the datasets Filters field, before applying the following variant and genotype filters; genotypes were retained if they had a depth (DP) ≥ 10, genotype quality score (GQ) ≥ 30, allelic balance (AB) < 0.1 in homozygous calls for the reference allele, AB ≥ 0.25 and ≤ 0.75 for heterozygous calls and AB ≥ 0.9 for homozygous calls for the alternative allele. Variants were excluded if they had a call rate across all samples < 0.9 or a Hardy–Weinberg Equilibrium exact test P value of < 1 × 10^−6^.

***Variant annotation***

Variants were annotated using Hail’s Ensembl Variant Effect Predictor (version 96) ^14^. We defined PTVs as variants annotated as stop-gain, frameshift and splice donor/acceptor variants. Damaging-missense variants were defined as missense variants with a “Missense badness, Polyphen-2, constraint” (MPC) score ≥ 2 ^15^, these are known to be associated with psychiatric and neurodevelopmental disorders^16,17^. For our primary analyses, we sought to enrich for pathogenic variants by restricting to ultra-rare variants, which we defined as those that are observed only once in our data set (i.e. a Minor Allele Count [MAC] of 1) and are not present among 114,704 individuals in the gnomAD non-neuro dataset (gnomAD v2.1.1) ^18^.

**eMethods 6**

***Polygenic Risk Scores and Copy Number Variants***

Standardised polygenic risk scores (PRS) were calculated using PRSice ^19^ following a widely applied method ^20^ using default parameters unless otherwise stated ^7^. The PRS for schizophrenia (SZ PRS) and intelligence (IQ PRS) were calculated for each participant based on summary statistics from the largest publicly available genome-wide association studies (GWAS) for these traits^21^. We ensured our schizophrenia sample was independent from the schizophrenia GWAS using genome based de-duplication. The first 5 genetic principal components were included as covariates when generating each PRS. As in a previous study ^7^, we generated PRS based on single-nucleotide variants associated with a threshold of P_T_ ≤ 0.05 in the schizophrenia and IQ GWAS. Finally, known schizophrenia associated CNV calls were generated as detailed in our previous publication ^22^.

**eMethods 7**

***Study design and statistics***

 We performed a within-case analysis of the relationship between the burden of rare coding variants and cognitive ability. Recent studies have shown that evidence of selective constraint, at either the variant or gene level, is a feature associated with rare coding variants that contribute to impaired cognition in people with schizophrenia^23^ or ASD^17^, as well as in the general population^24^. Thus, in the current study, we postulated that “ultra-rare, constrained variants” (URCVs), defined as either PTVs in LoFI genes (genes with gnomAD “probability of loss-of-function intolerance” (pLI) scores ≥ 0.9 ^25^) or damaging-missense variants (MPC ≥ 2) ^26^ that are observed once in our sample and are not present in the gnomAD non-neuro dataset, are associated with lower measures of current cognition in people with schizophrenia. We next investigated the effects of URCVs on estimated premorbid IQ to assess whether these effects explained the associations between URCVs and current cognition.

Additionally, given that cognitive impairment is a usual characteristic of NDDs, we postulated that cognitive ability would be impaired in carriers of ultra-rare constrained variants in a set of 348 genes previously implicated in neurodevelopmental disorders (NDD) including schizophrenia ^17,27,28^. We call this set of genes, NDD genes.

We used linear regression to test for association between cognition and the number of rare coding variants carried by each individual, with variants defined using the above criteria. We covaried for sex, age at interview, sequencing site, principal components 1-10, and exome wide burden of synonymous variants meeting the same allelic frequency threshold as the test set of variants. Of the 802 individuals that passed QC, 754 had measures of premorbid IQ, 762 of current cognition, and 721 for both.

In previous work, we showed that rare deletion CNVs affecting LoFI genes, SZ PRS and IQ PRS are associated with lower cognitive ability in people with schizophrenia ^7,22^. Here, we performed a multivariable analysis of the effects of rare coding variants, SZ PRS, IQ PRS and SZ CNVs on cognitive ability, including each of these as predictors in a linear regression model.

**eFigure 7:** **Density plots showing the distribution of premorbid IQ and current cognition scores for carriers and non-carriers of ultra-rare, constrained variants.**

****A**. Distribution of premorbid IQ scores across carriers and non-carriers of ultra-rare, constrained variants. **B**. Distribution of current cognition scores across carriers and non-carriers of ultra-rare, constrained variants. N = non-carriers of ultra-rare, constrained variants, Y = carriers of ultra-rare, constrained variants. The dashed vertical lines show the mean cognitive score for carriers and non-carriers.

**eResults**

***Covarying for diagnosis***

As our sample contained different schizophrenia related diagnoses (schizophrenia, schizoaffective disorder, schizophreniform psychoses and other psychotic disorders), we evaluated whether there was an effect of diagnosis on cognitive function. Including a covariate for diagnosis in our primary analysis of URCVs did not impact our findings shown in table 1 of the main text, and below in **eTable 3** (*p =* 0.005 to *p* = 0.006).

| **eTable 3: Association between URCVs and cognition when covarying for diagnosis.**  URCVs, defined as those with PTVs in loss-of-function intolerant genes and damaging missense variants (MPC ≥ 2), were evaluated to see if they were associated premorbid IQ in 754 participants and current cognition in 762 participants with schizophrenia and related psychotic disorders when controlling for diagnosis. The effect size is the β estimate from our linear model and represents the change in cognitive measure associated with the presence of a URCV. | | | | | |
| --- | --- | --- | --- | --- | --- |
| **Test** | **Variants** | **Cognitive Measure** | **Effect Size** | **SE** | ***P-Value*** |
| URCVs | 392 | Premorbid IQ | -0.12 | 0.05 | 0.02 |
|  | 400 | Current Cognition | -0.18 | 0.07 | 0.005 |
| URCVs  (covarying for diagnosis) | 392 | Premorbid IQ | -0.12 | 0.05 | 0.03 |
|  | 400 | Current Cognition | -0.18 | 0.06 | 0.006 |

***Sensitivity Analysis***

We performed a sensitivity analysis of our primary analysis by annotating PTVs as either “high” or “low” confidence using LOFTEE^18^. When excluded low confidence PTVs from the analysis we saw a trivial reduction in effect size (**eTable 4**) and a reduction of the number of variants (n = 95).

| **eTable 4: Evaluating the impact of removing LOFTEE low-confidence PTVs on the association between URCVs and cognition in schizophrenia.**  URCVs, defined as those with PTVs in loss-of-function intolerant genes and damaging missense variants (MPC ≥ 2), were evaluated to see if they were associated current cognition in 762 participants with schizophrenia and related psychotic disorders. The effect size is the β estimate from our linear model and represents the change in cognitive measure associated with the presence of a URCV. | | | | | | |
| --- | --- | --- | --- | --- | --- | --- |
| **Cognitive Measure** | **N Variants** | **LOFTEE low-confidence PTVs removed** | **Effect Size** | **SE** | ***P-Value*** |  |
| Current Cognition | 400 | No | -0.18 | 0.07 | 0.005 |  |
|  | 305 | Yes | -0.17 | 0.08 | 0.02 |  |

***Independent effects of missense variants and PTVs***

We evaluated the independent effects of ultra-rare damaging missense variants (MPC ≥ 2) and ultra-rare PTVs in loss-of-function intolerant genes on cognition. While the effect of damaging-missense variants on both premorbid and current cognition was larger than for PTVs, the difference in effect size between the two classes of mutation was not significant when tested using a Z-test (**eTable 5**).

| **eTable 5: Independent effects of damaging missense variants (MPC ≥ 2) and PTVs on cognition.**  The association between the independent effects of ultra-rare damaging missense variants or ultra-rare PTVs in loss-of-function intolerant genes on premorbid IQ in 754 participants and current cognition in 762 participants with schizophrenia and related psychotic disorders. The effect size is the β estimate from our linear model and represents the change in cognitive measure associated with the presence of either an MPC ≥ 2 or PTV in LoFI gene. | | | | | | |
| --- | --- | --- | --- | --- | --- | --- |
| **Test** | **Variants** | **Cognitive Measure** | **Effect Size** | **S**E | ***P-Value*** | **Z Test P** |
| **MPC ≥ 2** | 256 | **Premorbid IQ** | -0.11 | 0.06 | 0.09 | 0.24 |
| **PTVs** | 136 |  | -0.12 | 0.08 | 0.15 |  |
| **MPC ≥ 2** | 260 | **Current Cognition** | -0.20 | 0.08 | 0.02 | 0.34 |
| **PTVs** | 140 |  | -0.14 | 0.10 | 0.16 |  |

***Effects of Duration of Disorder and Age of Onset on Current Cognition***

We investigated whether duration of disease and age of onset could be attributed to effect of URCVs on current cognition covarying for premorbid IQ. We did this by regressing current cognition on age at interview, and the residuals from this were used as the outcome variables in a multivariable linear regression on the covariates including duration. This allowed us to avoid any issues with co-linearity in the model and to determine that there was not attributable effects of duration of illness or age at onset on current cognition (**eTable 6**).

| **eTable 6: Effects of duration of disorder and age at onset on current cognition corrected for age at interview.**  Current cognition was regressed on age at interview, and the residuals from this were used as the outcome variables in a multivariable linear regression on the covariates shown in the table. | | | |
| --- | --- | --- | --- |
| **Test** | **Effect Size** | **Std. Error** | ***P-Value*** |
| URCVs | -0.09 | 0.05 | 0.07 |
| Duration of Disorder | -0.01 | 0.003 | 0.001 |
| Age of Onset | 0.002 | 0.004 | 0.63 |
| Premorbid IQ | 0.57 | 0.03 | <0.0001 |
| Sex | 0.19 | 0.07 | 0.005 |
| Synonymous Variants | 0.02 | 0.01 | 0.07 |

***Current cognition - Domain specific analysis***

Current cognition is our MATRICS composite z-score (ZComposite score) derived from domain *z scores* using mean and standard deviations of 103 healthy controls also recruited as part of the Cardiff COGs study, following the MCCB manual procedures protocol ^6^. The MATRICS cognitive composite z-score was calculated as described previously ^7^. Premorbid IQ was estimated using the National Adult Reading Test (NART) ^8^ and is correlated with specific aspects of the ZComposite score. We tested the association between URCVs and each domain and found the strongest effects coming from those domains most highly correlated with premorbid IQ (**eTable 7**).

| **eTable 7: Specific domains of current cognition and their association with URCVs.**  Current cognition is a ZComposite score made up from scores generated across 7 domains. This table looks at the association between URCVs and each domain in 762 participants with schizophrenia and related psychotic disorders. It also shows the correlation between each domain and premorbid IQ (measured by NART) and current cognition (ZComposite Score). The effect size is the β estimate from the linear regression and corresponds to the difference in standardised cognition measure associated with each URCV carried. | | | | | | | |
| --- | --- | --- | --- | --- | --- | --- | --- |
| **Test** | **Variants** | **Cognitive Measure** | **Cor. premorbid IQ** | **Cor. with Current Cognition** | **Effect Size** | **Std. Error** | ***P-Value*** |
| Constrained Variants | 400 | Problem Solving (Mazes) | 0.26 | 0.73 | -0.12 | 0.06 | 0.05 |
|  |  | Verbal Learning | 0.43 | 0.80 | -0.16 | 0.08 | 0.04 |
|  |  | Visual Learning | 0.39 | 0.80 | -0.09 | 0.06 | 0.12 |
|  |  | Attention | 0.43 | 0.78 | -0.17 | 0.06 | 0.01 |
|  |  | Speed of Processing | 0.37 | 0.84 | -0.12 | 0.05 | 0.02 |
|  |  | Working Memory | 0.52 | 0.84 | -0.18 | 0.06 | 0.003 |
|  |  | Social Cognition | 0.25 | 0.52 | -0.02 | 0.06 | 0.71 |

***URCVs, CNVs, schizophrenia PRS, IQ PRS and cognitive function.***

In order to get a more complete picture of the relationship between genomic variation and the independent effects of these four classes of variants: URCVs, CNVs (**eTable 8**) and schizophrenia PRS or IQ PRS and cognition in schizophrenia, we performed a series of **univariate** analyses (**eTable 9**).

| **eTable 8: Schizophrenia associated copy number variants identified in the Cardiff COGS cohort.**  WBS = Williams-Beuren syndrome. | | |
| --- | --- | --- |
| **CNV** | **Coordinates (build 37)** | **Number of carriers** |
| 1q21.1 duplication | 1:146527987-147394444 | 1 |
| *NRXN1* deletion | 2:50145643-51259674 | 1 |
| WBS duplication | 7:72744915-74142892 | 1 |
| 15q11.2 deletion | 15:22805313-23094530 | 3 |
| 16p13.11 duplication | 16:15511655-16293689 | 3 |
| 16p12.1 deletion | 16:21950135-22431889 | 2 |
| 16p11.2 duplication | 16:29650840-30200773 | 1 |
| 22q11.2 deletion | 22:19037332-21466726 | 2 |

For premorbid IQ, CNVs had the largest effect size followed by IQ PRS and then URCVs, although there is substantial imprecision in the estimate of effect size for CNVs. Similar patterns were obtained for current cognition.

| eTable 9: Univariable analyses for the independent impact on cognition of URCVs, schizophrenia (SZ), IQ PRS and CNV carrier status.  URCVs, SZ PRS, IQ PRS and CNV carrier status were tested individually using a univariable linear regression model to evaluate their effects upon premorbid IQ in 679 participants and current cognition in 648 participants with schizophrenia or related psychotic disorders. URCVs, SZ PRS, IQ PRS and CNV carrier status were tested individually using a univariable linear regression model to evaluate their effects on premorbid IQ in 679 participants and current cognition in 648 participants with schizophrenia or related psychotic disorders. The effect size is the β estimate from the linear regression and corresponds to the difference in standardised cognition measure associated with each URCV carried, or an increase of 1 standard deviation of the PRS. | | | | |
| --- | --- | --- | --- | --- |
| Cognitive Measure | **Test** | **Univariable analysis** | | |
|  |  | **Effect Size** | **SE** | ***P-Value*** |
| Premorbid IQ | URCVs | -0.12 | 0.05 | 0.02 |
|  | SZ PRS | -0.01 | 0.04 | 0.80 |
|  | IQ PRS | 0.30 | 0.04 | <0.0001 |
|  | CNV | -0.72 | 0.28 | 0.01 |
| Current Cognition | URCVs | -0.18 | 0.07 | 0.01 |
|  | SZ PRS | -0.12 | 0.05 | 0.02 |
|  | IQ PRS | 0.30 | 0.05 | <0.00001 |
|  | CNV | -0.65 | 0.37 | 0.08 |

***eReferences***

1. Rehman F. Schedules for clinical assessment in neuropsychiatry. *BMJ*. 2011;342(6):589-593. doi:10.1136/BMJ.C7160

2. Wing JK, Babor T, Brugha T, et al. SCAN: Schedules fonr Clinical Assessment in Neuropsychiatry. *Archives of General Psychiatry*. 1990;47(6):589-593. doi:10.1001/ARCHPSYC.1990.01810180089012

3. American Psychiatric Association. Diagnostic and Statistical Manual of Mental Disorders (4th edn). 1994;DSM-IV.

4. Lynham AJ, Hubbard L, Tansey KE, et al. Examining cognition across the bipolar/schizophrenia diagnostic spectrum. *Journal of Psychiatry and Neuroscience*. 2018;43(4):245-253. doi:10.1503/JPN.170076

5. Green MF, Nuechterlein KH. The MATRICS initiative: Developing a consensus cognitive battery for clinical trials. *Schizophrenia Research*. 2004;72(1):1-3. doi:10.1016/J.SCHRES.2004.09.006

6. Nuechterlein KH, Green MF, Kern RS, et al. The MATRICS consensus cognitive battery, part 1: Test selection, reliability, and validity. *American Journal of Psychiatry*. 2008;165(2):203-213. doi:10.1176/APPI.AJP.2007.07010042

7. Legge SE, Cardno AG, Allardyce J, et al. Associations Between Schizophrenia Polygenic Liability, Symptom Dimensions, and Cognitive Ability in Schizophrenia. *JAMA Psychiatry*. Published online August 2021. doi:10.1001/JAMAPSYCHIATRY.2021.1961

8. Nelson HE, Willison J. *The National Adult Reading Test (NART)*. NFER-Nelson; 1991.

9. McKenna A, Hanna M, Banks E, et al. The Genome Analysis Toolkit: a MapReduce framework for analyzing next-generation DNA sequencing data. *Genome research*. 2010;20(9):1297-1303. doi:10.1101/GR.107524.110

10. Li H, Durbin R. Fast and accurate short read alignment with Burrows-Wheeler transform. *Bioinformatics*. 2009;25(14):1754-1760.

11. Pedersen BS, Quinlan AR. Mosdepth: quick coverage calculation for genomes and exomes. *Bioinformatics*. 2018;34(5):867-868. doi:10.1093/BIOINFORMATICS/BTX699

12. Pedersen BS, Bhetariya PJ, Brown J, et al. Somalier: Rapid relatedness estimation for cancer and germline studies using efficient genome sketches. *Genome Medicine*. 2020;12(1):1-9. doi:10.1186/s13073-020-00761-2

13. Conomos MP, Reiner AP, Weir BS, Thornton TA. Model-free Estimation of Recent Genetic Relatedness. *American Journal of Human Genetics*. 2016;98(1):127-148. doi:10.1016/j.ajhg.2015.11.022

14. McLaren W, Gil L, Hunt SE, et al. The Ensembl Variant Effect Predictor. *Genome Biology*. 2016;17(1):1-14. doi:10.1186/s13059-016-0974-4

15. Samocha KE, Kosmicki JA, Karczewski KJ, et al. Regional missense constraint improves variant deleteriousness prediction. *bioRxiv*. Published online June 2017:148353-148353. doi:10.1101/148353

16. Singh T, Poterba T, Curtis D, et al. Rare coding variants in ten genes confer substantial risk for schizophrenia. *Nature*. 2022;604(7906):509-516. doi:10.1038/s41586-022-04556-w

17. Satterstrom FK, Kosmicki JA, Wang J, et al. Large-Scale Exome Sequencing Study Implicates Both Developmental and Functional Changes in the Neurobiology of Autism. *Cell*. 2020;180(3):568-584.e23. doi:10.1016/j.cell.2019.12.036

18. Karczewski KJ, Francioli LC, Tiao G, et al. The mutational constraint spectrum quantified from variation in 141,456 humans. *Nature 2020 581:7809*. 2020;581(7809):434-443.

19. Euesden J, Lewis CM, O’Reilly PF. PRSice: Polygenic Risk Score software. *Bioinformatics*. 2015;31(9):1466-1468. doi:10.1093/BIOINFORMATICS/BTU848

20. Wray NR, Lee SH, Mehta D, Vinkhuyzen AAE, Dudbridge F, Middeldorp CM. Research Review: Polygenic methods and their application to psychiatric traits. *Journal of Child Psychology and Psychiatry and Allied Disciplines*. 2014;55(10):1068-1087. doi:10.1111/JCPP.12295

21. The Schizophrenia Working Group of the Psychiatric Genomics Consortium, Ripke S, Walters JT, O’Donovan MC. *Mapping Genomic Loci Prioritises Genes and Implicates Synaptic Biology in Schizophrenia*. Genetic and Genomic Medicine; 2020. doi:10.1101/2020.09.12.20192922

22. Hubbard L, Rees E, Morris DW, et al. Rare Copy Number Variants Are Associated With Poorer Cognition in Schizophrenia. *Biological Psychiatry*. 2021;90(1):28-34. doi:10.1016/J.BIOPSYCH.2020.11.025

23. Singh T, Walters JTRR, Johnstone M, et al. The contribution of rare variants to risk of schizophrenia in individuals with and without intellectual disability. 2017;49(8):1167-1173. doi:10.1038/ng.3903

24. Gardner EJ, Neville MDC, Samocha KE, et al. Sex-biased reduction in reproductive success drives selective constraint on human genes. *bioRxiv*. Published online December 2020:2020.05.26.116111-2020.05.26.116111. doi:10.1101/2020.05.26.116111

25. Karczewski KJ, Francioli LC, Tiao G, et al. Variation across 141,456 human exomes and genomes reveals the spectrum of loss-of-function intolerance across human protein-coding genes. *bioRxiv*. 2019;19(5):531210-531210. doi:10.1101/531210

26. Samocha K, Kosmicki J, Karczewski K, et al. Regional missense constraint improves variant deleteriousness predictio. Published online 2017. doi:10.1101/148353

27. Singh T, Neale BM, Daly MJ. Exome sequencing identifies rare coding variants in 10 genes which confer substantial risk for schizophrenia. *medRxiv*. Published online September 2020. doi:10.1101/2020.09.18.20192815

28. Kaplanis J, Samocha KE, Wiel L, et al. Evidence for 28 genetic disorders discovered by combining healthcare and research data. *Nature 2020 586:7831*. 2020;586(7831):757-762.
